# Hierarchical CoMn-LDH and Heterostructured Composites for Advanced Supercapacitors and Electrocatalysis Applications

**DOI:** 10.3390/ma18030604

**Published:** 2025-01-28

**Authors:** Ganesh T. Chavan, Deepak P. Dubal, Pritam J. Morankar, Chan-Wook Jeon, Jinsung An, Ki-Han Song

**Affiliations:** 1 Department of Civil & Environmental Engineering, Hanyang University ERICA, Ansan 15588, Republic of Korea; gtchavan1992@gmail.com (G.T.C.); jsan86@hanyang.ac.kr (J.A.); 2 Centre for Materials Science, School of Chemistry and Physics, Queensland University of Technology (QUT), 2 George Street, Brisben, QLD 4000, Australia; 3 School of Chemical Engineering, Yeungnam University, 280 Daehak-ro, Gyeongsan 38541, Republic of Korea; 4 Department of Civil Engineering, Seoul National University of Science and Technology, Seoul 01811, Republic of Korea

**Keywords:** bifunctional electrode, hydride supercapacitor device, cyclic stability, overpotential, Tafel slope

## Abstract

In the present study, self-assembled hierarchical CoMn-LDH, CoMn@CuZnS, and CoMn@CuZnFeS heterostructured composites were synthesized for bifunctional applications. As an electrode for a supercapacitor, CoMn-LDH demonstrated superior areal and specific capacitance of 5.323 F cm^−2^ (279.49 mAh/g) at 4 mA cm^−2^, comparable to or even higher than other LDHs. The assembled AC//CoMn-LDH hybrid supercapacitor device further demonstrated better stability with 63% original capacitance over 20,000 cycles. Later, as a catalyst, CoMn-LDH, CoMn@CuZnS, and CoMn@CuZnFeS electrodes revealed better performance, with overpotentials of 340, 350, and 366 and −199, −215, and −222 mV to attain 10 mA cm^−2^ of current density for the oxygen evolution reaction (OER) and hydrogen evolution reaction (HER), respectively. Moreover, for CoMn-LDH, small Tafel slopes of 102 and 128 mV/dec were noticed for OER and HER with good stability compared to heterostructured electrodes.

## 1. Introduction

Modern society faces significant challenges posed by the energy crisis, global warming due to excessive CO_2_ emissions, and severe health threats, all of which are caused by the large-scale utilization of fossil fuels [1,2,3,4,5]. Hence, the exploration of novel, clean, and efficient energy storage technologies has become essential [1,2,3,4]. In this ongoing effort to switch to green energy, supercapacitors (SCs) are considered a promising technology [6,7]. The excellent properties of SCs, namely fast charging and slow discharging rates, flexibility, long-term stability, high power density (PD), exceptional rate capability, absence of the memory effect, and environmental friendliness, suggest that SCs are suitable candidates for use in various high-tech applications. For example, SC devices are in demand for applications such as buses, trains, cranes, biomedical devices, memory backups, automobiles, and aircraft [6,7,8]. However, the widespread commercialization of SCs has been limited by their poor energy density (ED), low conductivity, and polarization effects [7,9]. To address these shortcomings, researchers have sought to replace the redox-active heterostructured electrode materials [6]. Recently, layered double hydroxides (LDHs) have gained significant attention as favorable SC materials owing to their abundance, cheap cost, large specific surface area, unique lamellar structure, and composition modulation capability [10]. In particular, for energy storage applications, LDH materials have several advantages: (i) LDHs occur in different morphologies, including microflowers, hollow spheres, nanoparticles, nanoflexes, hollow spheres, nanoparticles, microflowers, nanorods, nanoplates, nanosheets, and nanotubes [11], which provide additional active sites and reduce the diffusion path length; (ii) LDHs show excellent anion exchange performance; and (iii) composition modulation in LDHs strongly influences the energy storage properties through the formation of different oxidation states, such as Co^2+^, Mn^2+^, and Ni^2+^ [11]. Different noteworthy approaches, such as composite design, nanostructure engineering, interface engineering, and composition optimization, have been implemented to address the issues associated with LDH materials [12]. Another important strategy is to design and develop heterostructured composite electrode materials that can exploit the interaction between different metal atoms to realize enhanced energy storage performance [13].

Due to its high ED and lack of adverse environmental effects, hydrogen is now considered the primary candidate fuel for replacing fossil fuels [14]. The most efficient approach for hydrogen production is electrocatalytic water splitting, which contains oxygen evolution reaction (OER) at the anode and hydrogen evolution reaction (HER) at the cathode [14]. Recently, intense attempts have been made to explore different electrocatalyst materials, such as oxides (or hydroxides), sulfides, transition metals, selenides, phosphides, phosphates, and nitrides [14,15]. Pt-based materials are considered ideal HER and OER catalysts. However, their widespread practical utilization is restricted by their expensive nature, poor stability, and methanol resistance [15,16]. Therefore, designing and developing inexpensive, efficient, and stable bifunctional electrocatalysts to achieve low overpotentials in alkaline media for OER and HER is an urgent need [16,17]. LDHs, denoted as [M^2+^_1−x_M^3+^_x(OH)2_] [A^n−^]_x/n_ ZH_2_O, have become popular electrocatalyst materials due to their unique electron distributions and layered structure [17]. Different bimetallic or trimetallic LDHs, such as Co-Fe, Ni-Co, Ni-V, Co-Co, Ni-Fe, Ni-Mn, Co-Cr, Co-Mn, Ni-Ni, Co-Ni, Ni-Co-Fe, and Ni-Mn-Co, have been widely explored as HER and OER electrocatalysts [17,18,19,20]. Consequently, new bimetallic LDH materials that address the shortcomings of the current LDHs while maintaining their advantageous features must be developed. Among various transition metal hydroxides, Co hydroxide exhibits excellent redox activity; however, the cost is high due to the low abundance of Co. By contrast, Mn is environmentally friendly and has a high theoretical capacitance in supercapacitor applications [21]. Combining or doping with Mn ions has several advantages, including regulating the electronic structure of the catalyst by Mn, providing additional active sites, and increasing the intrinsic catalytic activity by weakening the interaction between H and Co atoms to regulate the free energy of H adsorption [22,23]. Thus, a CoMn-LDH electrode formed from a combination of manganese hydroxide and cobalt hydroxide is expected to show improved energy storage and catalytic performance. In this context, bimetallic CoMn-LDH is recognized as a leading candidate for obtaining enhanced electrocatalytic activity because of its fascinating characteristics, such as low cost, environmental friendliness, excellent physicochemical properties, high redox activity, high oxidation potential for Co and Mn, and multiple oxidation states [19,24]. In recent studies, several researchers have reported on CoMn-LDH electrodes for the fabrication of hybrid supercapacitor devices (HSCs), including nanoflake-like CoMn-LDH//activated carbon (AC) electrodes [24], electrodeposited CoMn-LDH for CoMn-LDH//AC [21], nanoflake-type CoMn-LDH//AC [25], and CoMn-LDH nanowire-based CoMn-LDH//AC [26]. Some studies have also explored CoMn-LDH electrodes for HER, OER, and overall water electrolysis applications. For example, Yan et al. discussed the use of CoMn-LDH nanosheets for OER [27], Pan et al. reported on a specific reduction–oxidation method for the growth of CoMn-LDH for OER [28], Bao et al. prepared an ultrathin nanosheet of CoMn-LDH for bifunctional electrocatalysts (OER and HER) [29], and Song et al. successfully deposited CoMn-LDH in the form of ultrathin nanoplates for OER application [30]. Unfortunately, volume changes, low electron transport rates, poor long-term stability, low electronic conductivity, and a limited number of electroactive sites lead to poor energy storage and unsatisfactory water-splitting performance of these materials [19,20]. Consequently, it is a great task to overwhelm their shortcomings without losing advantages. To surpass the high catalytic reaction barrier, decoration of 1D, 2D, or 3D materials on 2D materials is recommended as a fruitful strategy to improve ion transport and interfacial charge distribution.

On the other hand, TM oxides/sulfides, such as AX_2_O_4_/AXS (A, X = Fe, Co, Zn, Mn, Cu, Ni, etc.), are gaining more interest as positive electrode materials in SC and water-splitting applications [31]. Moreover, in TMS/TMO, metal ions (Fe, Cu, and Zn) play a crucial role in enhancing the electrical conductivity and multiple valence states, and the synergistic impact of the multi-component system helps to promote electrochemical activity. Thus, by selecting a proper ratio of divalent ions, spinal metal ferrites catalytically activate the electrode surface [31,32]. In this context, binary CuS and ZnS compounds are attractive contenders for bifunctional application [31]. Specifically, CuFeS_2_/CuFeO_2_ structures are considered promising because of admirable electrochemical characteristics and high conductivity originating from the tetrahedral coordination with sulfur and systematic adjustment of Fe and Cu ions in the crystal matrix. CuFeO_2_ electrodes with a double sphalerite cell structure deliver a capacitance of 128 F g^−1^ with 96% retention over 10,000 cycles [33]. Ranjani et al. described that a symmetric device assembled by CuFeS_2_ samples revealed a specific power and energy of 1146 W kg^−1^ and 16 Wh kg^−1^, respectively, with a 94% fade in capacity after 6000. The encouraging electrochemical performance is supported by the synergistic impact of Fe^3+^ and Cu^+^ ions in the redox process [33]. Moreover, chemically synthesized CoZnS exhibits a capacitance of 1288 Fg^−1^ at 2 mA cm^−2^ and depicts an 85% decline in capacity over 5000 cycles [7]. The CuMnZnS@NF electrodes reveal a remarkable capacitance of 9.56 F cm^−2^ and 70% preserved capacity over 5000 cycles [31]. Furthermore, Zn and Cu have similar ionic sizes (Zn^2+^ = 0.74 Å and Cu^2+^ = 0.72 Å) [34], which, when combined, are expected to increase the active sites. In this scenario, one effective strategy is the development of a well-designed hierarchical heterostructure with unique morphologies and improved physicochemical properties that can offer extra electroactive sites, interfacial electronic interaction, increased electrical conductivity, a large surface area, improved surface reaction kinetics, and fast charge transfer, promoting surface redox reactions. Hence, for the first time, we decorated CuZnS and CuZnFeS nanostructures on CoMn-LDHs to form CoMn@CuZnS and CoMn@CuZnFeS heterostructured composites for improved supercapacitive and electrocatalytic performance. Almost no or only a few studies have been carried out on CoMn@CuZnS, and CoMn@CuZnFeS heterostructures as efficient bifunctional catalysts and for application in high-energy-density hybrid supercapacitors, where it is used as a material for the positive electrode and AC is utilized for the negative electrode. Therefore, the current investigation aims to explore the engineering of LDH-based heterostructured composites to advance ion or electron transport to achieve improved energy storage performance and superior bifunctional catalysis.

The physical characteristics and energy storage performance of the CoMn-LDH, CoMn@CuZnS, and CoMn@CuZnFeS heterostructures were carried out by different techniques. The structural assessment of the CoMn-LDH, CoMn@CuZnS, and CoMn@CuZnFeS heterostructures was conducted by XRD measurement. XPS measurement was carried out to identify the oxidation states in CoMn-LDH electrodes. Morphology and material compositions of the as-deposited heterostructures were observed by employing FESEM and EDS techniques. Furthermore, the supercapacitive characteristics were verified via three-electrode configurations, which included cyclic voltammetry and charge–discharge measurements, to investigate and determine the different performance parameters. The electrocatalytic measurements were performed to record linear sweep voltammetry and chronopotentiometry for the analysis of HER and OER performance. The charge transfer resistance associated with the heterostructures was measured with the help of electrochemical impedance spectroscopy. Furthermore, an AC//CoMn-LDH HSC device was fabricated using AC as the anode and as-deposited CoMn-LDH as the cathode. The AC//CoMn-LDH HSC device exhibited remarkable energy storage capacity. Furthermore, the small Tafel slopes, low overpotentials for OER and HER, and promising stability in alkaline environments indicated the superior bifunctional catalytic performance of CoMn-LDH, CoMn@CuZnS, and CoMn@CuZnFeS heterostructures.

## 2. Materials and Methods

### 2.1. Synthesis of CoMn-LDH

CoMn-LDH was deposited onto porous nickel foam (NF) substrates using a solvothermal method. Initially, cobalt (II) chloride hexahydrate (CoCl_2_ 6H_2_O, 3 mM) and manganese (II) chloride (MnCl_2_, 5 mM) were mixed in deionized (DI) water (50 mL). Then, ammonium fluoride (NH_4_F, 2 mM) and urea (NH_2_CONH_2_, 9 mM) were mixed into the above solution. A uniform precursor solution was formed via vigorous stirring. Then, NF substrates (5 cm × 1 cm) and precursor solution were placed in an 80 mL Teflon liner, succeeded by heating at 130 °C for 4 h. After synthesis, the electrodes were washed with DI water and cooled to 27 °C before desiccation in a vacuum oven for 12 h at 60 °C. Physical characterization, electrochemical measurements, and different performance parameters were calculated using standard equations [5,35,36,37] and are included in the Supplementary Information (SI). Furthermore, details regarding the preparation of the activated carbon electrode and the assembling of AC//CoMn-LDH HSC are given in SI.

### 2.2. Synthesis of CoMn@CuZnS and CoMn@CuZnFeS Heterostructures

**Scheme 1:**
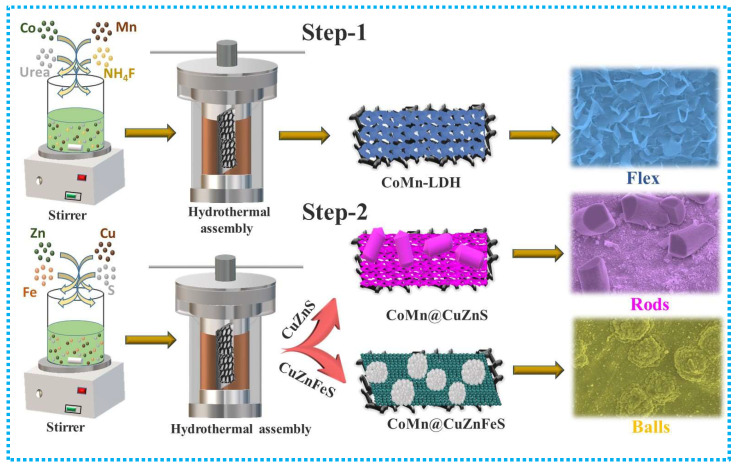
Schematic for synthesizing self-assembled CoMn-LDH, CoMn@CuZnS, and CoMn@CuZnFeS heterostructured composites.

The CuZnS and CuZnFeS composites were grown on CoMn-LDH base by the solvothermal technique. Copper (II) chloride dihydrate (CuCl_2_2H_2_O), zinc chloride (ZnCl_2_), iron (III) chloride hexahydrate (FeCl_3_6H_2_O), and thiourea (NH_2_CSNH_2_) (4 mM each) were distributed and continuously stirred to form a uniform solution in 50 mL of DI water. The as-prepared CoMn-LDH/NF and precursor solution were moved into a Teflon liner followed by heating at 130 °C for 2 h. The acquired samples were dried after being cleansed with DI water overnight at 60 °C. Scheme 1 displays the graphical illustration of LDH and the heterostructure growth process.

## 3. Results and Discussion

**Figure 1:**
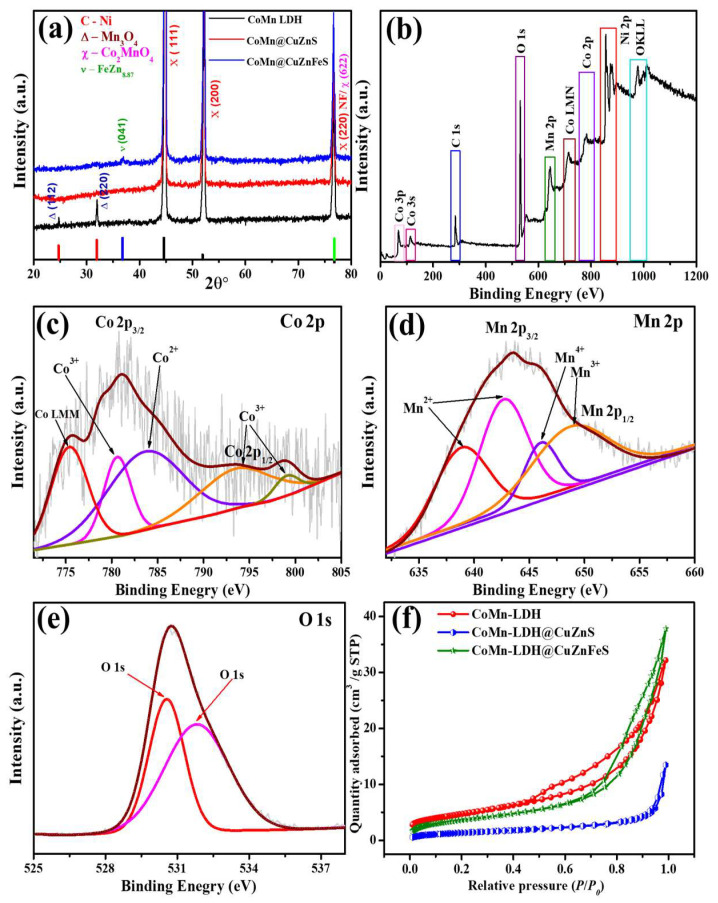
(**a**) XRD diffractograms of CoMn-LDH, CoMn@CuZnS, and CoMn@CuZnFeS heterostructures; (**b**) core-level XPS spectra; (**c**) Co 2p, (**d**) Mn 2p, and (**e**) O 1s for the CoMn-LDH electrode; and (**f**) BET plots of CoMn-LDH, CoMn@CuZnS, and CoMn@CuZnFeS samples.

Figure 1a depicts XRD plots of CoMn-LDH and the heterostructures. As shown in Figure 1a, there were three prominent reflections at 2θ of 44.856°, 52.200°, and 76.921° and d values of (111), (200), and (220), corresponding to the cubic Ni-phase, which agree with standard data (JCPDS # 00-001-1260). These Ni reflections are likely due to the NF substrate. The other two minor planes were found at d = 3.595 Å and 2.797 Å and 2θ of 24.765° and 31.991°, related to the Mn_3_O_4_ phase (JCPDS # 01-075-1560, 00-044-1472). Further, the FeZn_8.87_ phase was found in the CoMn@CuZnFeS sample at 2θ of 36.892° and d = 2.436 Å (JCPDS # 00-045-1185). Interestingly, the (220) reflection of Ni at 2θ = 76.921° was also a perfect match with the standard JCPDS data for the Co_2_MnO_4_ phase, with the (622) reflection indexed in Figure 1a (JCPDS # 00-001-1130). Therefore, XRD analysis revealed that all samples had low crystallinity or were nearly amorphous.

The XPS survey spectrum indicated the appearance of Co 2p, Mn 2p, and O 1s in the CoMn-LDH sample (Figure 1b). Figure 1c demonstrates the split peaks of Co 2p at ~781 and ~798 eV, which are related to Co 2p_3/2_ and Co 2p_1/2_, respectively [38,39]. Co 2p was split into four different peaks at 780.62, 793.74, 798.60, and 783.76 eV, which can be accredited to Co^3+^ and Co^2+^, implying the formation of CoMn-LDH [7,19]. The peak at 775.33 eV in the Co 2p core-level spectrum can be allocated to CoLMM [40]. The Mn 2p spectrum contained two peaks at 638.86 and 642.72 eV, analogous to the Mn (II) species, as seen in Figure 1d [41]. The other two peaks in the Mn 2p spectrum at 649.30 and 646.21 eV can be assigned to Mn^3+^ and Mn^4+^, respectively [42,43]. A plausible reason for the existence of Mn (IV) species is the lower oxidation and chemical stability of the Mn (III) state [42,43]. Thus, the XPS spectra of Mn 2p showed the presence of the Mn (II) and Mn (III) states in the form of Mn(OH)_2_ in CoMn-LDH [44]. Furthermore, the O 1s spectra, shown in Figure 1e, revealed two peaks at 530.55 and 531.81 eV, which can be attributed to the metal hydroxyl groups (Co-OH and Mn-OH), confirming the formation of CoMn-LDH material [44,45].

To assess the surface area and porosity of the CoMn-LDH, CuZnS, and CuZnFeS heterostructures, BET measurement was carried out, and Figure 1f reveals the adsorption-desorption curves. The specific surface area was 16.11, 8.82, and 12.62 m^2^/g for CoMn-LDH, CoMn@CuZnS, and CoMn@CuZnFeS heterostructures, respectively. Interestingly, CoMn-LDH exhibited a higher surface area than the other composites owing to the architecture of self-assembled, cross-linked, flexible nanosheets. Moreover, Table S1 in Supplementary Information (SI) displays different surface-related parameters, such as single point surface area, pore size, average pore volume, etc., for CoMn-LDH and other heterostructures.

**Figure 2:**
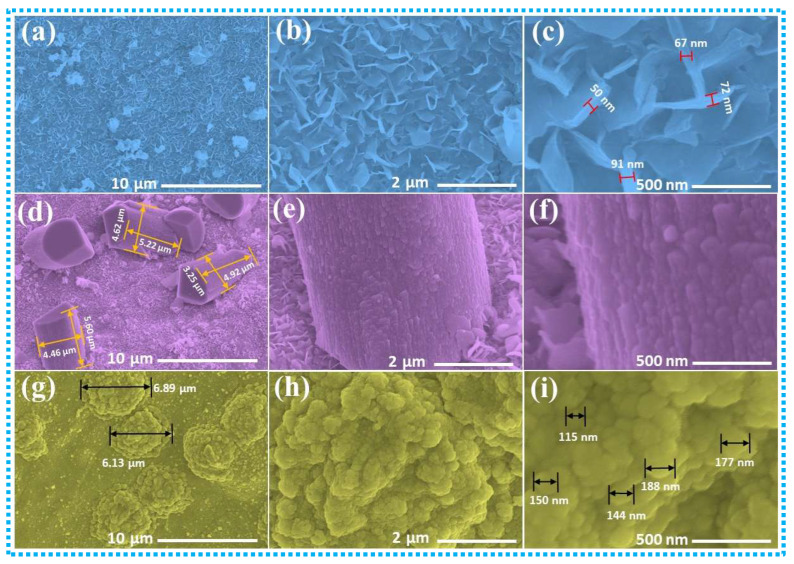
(**a**–**i**) High and low-magnification FESEM images of (**a**–**c**) CoMn-LDH, (**d**–**f**) CoMn@CuZnS, and (**g**–**i**) CoMn@CuZnFeS samples.

The FESEM images of CoMn-LDH and the heterostructures are shown in Figure 2a–i. CoMn-LDH showed uniformly grown, cross-linked, flex-type nanosheets covering the substrates (Figure 2a–c). The thickness of these flex-type nanosheets was approximately 50–100 nm. This type of 3D structure is considered a good framework for use as an electrode material for supercapacitors. Interestingly, such cross-linked nanosheets with large interspaces and high surface areas offer rich active sites for charge transport, making CoMn-LDH an ideal template for the growth of other redox-active materials [19,39]. The coating of CuZnS material on CoMn-LDH formed 3D road-like structures, which were randomly dispersed on the already grown core (flex-type nanosheets). Figure 2d–f reveals the diameter and height of the as-grown 3D rods, ranging from ~3.25–4.62 µm and ~4.9–5.6 µm, respectively. Notably, the 3D rods were dense and randomly distributed on a porous, flex-type core; hence, the overall porosity decreased. Some 3D rods looked like vertically planted tree stumps/trunks on the porous background (Figure 2e). Further, the deposition of CuZnFeS on CoMn-LDH changed the morphological appearance (Figure 2g–i). The porous flexes were completely covered by tiny circular granules with a reduction in total porosity. The low-resolution FESEM image (Figure 2g) showed the growth of rounded mounds of ~6–7 µm size. These rounded mounds were formed by tiny nanogranules having a size of ~100–200 nm. Overall, morphological observations suggested that the coating of CuZnS and CuZnFeS heterostructures on CoMn-LDH resulted in a reduction in the overall porosity of the surface, as already noticed in BET analysis.

**Figure 3:**
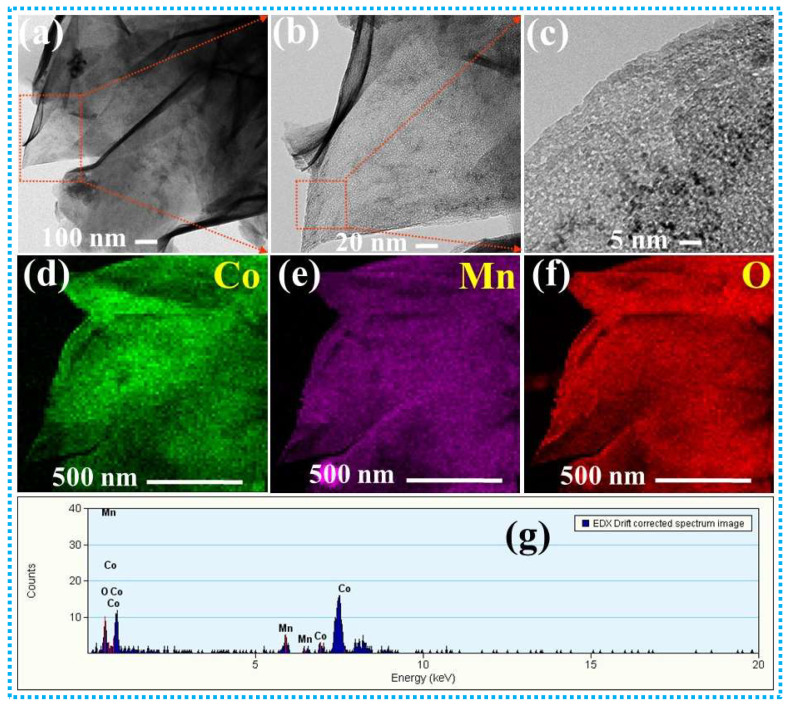
(**a**–**c**) TEM and HRTEM images; EDS mapping of (**d**) Co, (**e**) Mn, and (**f**) O; and (**g**) EDS plot of CoMn-LDH electrode.

Figure 3a–c shows low- and high-magnification (100 nm to 5 nm) TEM images of the CoMn-LDH sample. Similar to the SEM images, flake-type nanosheets were found in the TEM illustration, as shown in Figure 3a–c. The HRTEM image (Figure 3c) revealed more open pores, which were dispersed on the nanosheet, providing rapid transmission and exchange of electrolyte ions and electrons during electrochemical activity. As shown in Figure 3c, there was no fringe pattern, demonstrating the amorphous nature of the CoMn-LDH electrode. Figure 3d–f displays the outcomes of HAADF-STEM analysis and EDS mapping of the elements O, Mn, and Co in the CoMn-LDH electrode. Typical EDS spectra are shown in Figure 3g. Figure S1a–c confirm the formation of CoMn-LDH and heterostructured composites.

### 3.1. Supercapacitive Properties of CoMn-LDH, CoMn@CuZnS, and CoMn@CuZnFeS Heterostructures

**Figure 4:**
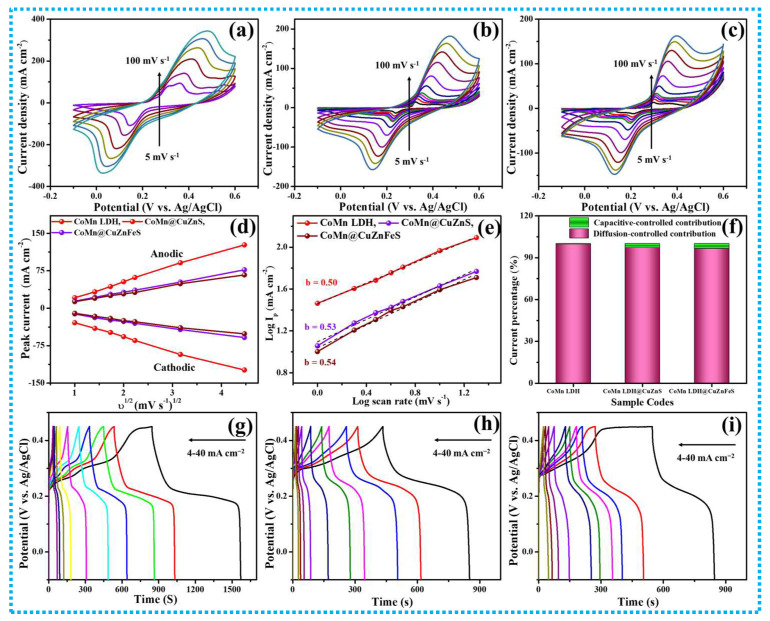
(**a**–**c**) CV, (**d**) i_p_ vs. v^1/2^, (**e**) log (i) vs. log (*υ*), and (**f**) ratios of diffusion and capacitive contribution and (**g**–**i**) GCD curves of CoMn-LDH, CoMn@CuZnS, and CoMn@CuZnFeS heterostructures.

To investigate the energy storage performance of CoMn-LDH, CoMn@CuZnS, and CoMn@CuZnFeS heterostructures, electrochemical studies were carried out using three electrodes in a solution of 4 M KOH. Voltage window optimization was carried out and is displayed in Figure S1d. Cyclic voltammetry (CV) and galvanostatic charge–discharge (GCD) graphs are shown in Figure 4a–c,g–i. All the samples displayed a pair of obvious redox peaks and a slight shift in the peak position, demonstrating quasi-reversibility, polarizability, and high conductivity, which enabled the ions and electrons to move quickly over the interface [8]. The Mn(OH)_6_ species might have provided additional reversible redox processes because of the OH^−^ ions from the electrolyte. However, with increasing scan rates, the CV profile remained unchanged, signifying the good capability of the samples. Insignificant changes in the CV plots suggest that good contact at the electrode/electrolyte interface aided the intercalation and de-intercalation processes at high scan rates. Nevertheless, decreased time for the charge–discharge process did not affect the intercalation and de-intercalation processes. The CV shape did not change with increased current density and specific capacity. Moreover, at high sweep rates, the electrodes displayed both diffusion-controlled and capacitive charge storage behaviors, possibly due to the M–OH/M–O–OH (where M denotes Co and Mn) redox transitions. The interaction between amorphous CoMn-LDHs was due to the redox reactions of the Co^2+^/Co^3+^, Mn^2+^/Mn^3+^, and Mn^3+^/Mn^4+^ states with OH^−^ anions [19], as described below:

(1) $$Mn{Co}_{2}O_{4}+H_{2}O+{OH}^{-}↔MnO{OH}^{-}+CoO{OH}^{-}+e^{-}$$

(2) $$CoOOH+{OH}^{-}↔CoO_{2}+H_{2}O+e^{-}$$

(3) $$MnOOH+{OH}^{-}↔MnO_{2}+H_{2}O+e^{-}$$

Remarkably, the coating of CoZnS and CoZnFeS composites on the CoMn-LDH nanosheets resulted in a lower peak current and area under the curve compared to pristine CoMn-LDH (Figure 4a–c). Hence, CV plots indicated that the coating of composite material resulted in a decrease in the charge storage capacity of CoMn-LDH owing to reduced porosity. The CV data at various scan speeds were thoroughly investigated to understand the charge storage kinetics and differentiate between the capacitive behavior and diffusion-controlled processes of electrodes. The Randles–Sevcik equation was employed to evaluate the diffusion coefficient (D) [5,46]:

(4) $$i_{p}=0.4463\times C\times A\times F\times n\;\;(\frac{nFDv}{RT}{)}^{1/2}$$

where υ is the sweep rate, and *C*, *D*, *i_p_*, *n*, *T*, *R*, *F*, and *A* are the electrolyte concentration, diffusion coefficient, peak current density, number of electrons, absolute temperature, universal gas constant, Faraday’s constant (96,485 C mol^−1^), and electrode area, respectively. For simplicity, Equation (4) is transformed as follows:

(5) $$\frac{i_{p}}{v^{1/2}}=2.69\times (10{)}^{5}\times C\times A\times (D{)}^{\frac{1}{2}}\times (n{)}^{\frac{1}{2}}\;\;$$

For the oxidation and reduction reactions, the D values of the CoMn-LDH, CoMn@CuZnS, and CoMn@CuZnFeS heterostructures for the oxidation process were found to be 1.149, 0.666, and 0.579 × 10^−7^ cm^3^ S^−1^, respectively (Figure 4d). A higher D value compared to heterostructures indicates higher ion mobility in the CoMn-LDH electrodes.

Dunn’s power law provides a quantitative correlation for the degree of capacitive impact [5,19]:

(6) $$i=av^{b}\;\;\;$$

where *a* and *b* are fitting variables. In this context, battery-type behavior plays a leading role when *b* = 0.5, whereas *b*~1 designates that the capacitive-controlled process is dominant [5,19,47]. Figure 4d,e reveals the plots of the peak current *i_p_* vs. v^1/2^ and log *i_p_* vs. log *υ*, respectively. Herein, the b value for the cathodic peak current was determined from Figure 4e and was found to be 0.5, 0.53, and 0.54 for CoMn-LDH, CoMn@CuZnS, and CoMn@CuZnFeS heterostructures, respectively. The b values of the electrodes indicate that the battery-type behavior played a major part in the charge storage process. Thus, the current response revealed a battery-like characteristic indicating Faradaic intercalation. The contributions of the battery type and capacitive behavior were quantitatively determined in terms of the overall charge stored. The overall charge stored (*Q_t_*) is the sum of the capacitive (*Q_s_*) and diffusion (*Q_d_*) contributions [5,48].

(7) $$Q_{t}=Q_{s}+Q_{d}\;\;\;$$

Moreover, the proportional contributions of both behaviors can be ascertained by employing the Bruce–Dunn approach [5,48]:

(8) $$i\left(V\right)=k_{1}v+k_{2}v^{1/2}\;\;\;$$

where υ is the scan rate, *i*(*V*) is the current, and *k*_1_ and *k*_2_ are constants. According to Equation (8), the redox reaction is defined by semi-infinite linear diffusion; therefore, *i*(*V*) changes linearly with *υ*^½^ for battery-type behavior, and *υ* changes directly for capacitive behavior. As a result, Equation (8) can be rearranged as follows [5,47]:

(9) $$\frac{i\left(V\right)}{v^{1/2}}=k_{1}v^{1/2}+k_{2}$$

Based on the slope and y-intercept attained by the linear fitting of the *i*(*V*)/*υ*^1/2^ vs. *v*^1/2^ plot, parameters *k*_1_ and *k*_2_ were determined for each electrode. The comparison of relative contributions of the two behaviors for all the electrodes at 5 mV s^−1^ is presented in Figure S1e–g and Figure 4f, and battery-type behavior was observed to be the major contributor to energy storage. The CoMn-LDH sample displayed ~99% battery-type contribution even up to a moderately fast sweep rate of (20 mV s^−1^), verifying the improved rate capability of CoMn-LDH.

The GCD plots of electrodes were obtained between −0.1 and 0.45 V in the voltage range with current densities of 4–40 mA cm^−2^ (Figure 4g–i). Additionally, all the electrodes showed nonlinear charge/discharge curves with clear characteristic plateaus, confirming classic battery-type behavior. Pseudocapacitive and battery-type materials were differentiated based on their CV and GCD curves. The reversible redox peaks in the pseudocapacitive materials were separated by a significant potential difference in the battery-type materials, and noticeable voltage plateaus were observed in the GCD curves during the GCD test. By contrast, the GDC curves of the pseudocapacitors did not exhibit voltage plateaus with symmetrical behavior in their CV plots [49]. Figure 4g–i shows that the discharge time decreased with growing current density (4 to 40 mA cm^−2^). Because the diffusion rate of alkali ions decreases with growing current density, only the electrode’s outer surface can be used for charge storage [50]. Furthermore, no significant IR drop in the GCD curves was observed, demonstrating that CoMn-LDH has a low internal resistance. The symmetric nature of the GCD plots demonstrated that the electrodes maintained a high Coulombic efficiency. CoMn-LDH exhibited a prolonged charge/discharge time, among other heterostructures, which is in agreement with the CV measurements. The C_A_ and Cs values were determined using standard equations (Equations (S1) and (S2)), as cited in Table S2, and were found to be 5.323 F cm^−2^ (279.49 mAh/g), 3.047 F cm^−2^ (150.17 mAh/g), and 2.210 F cm^−2^ (105.22 mAh/g) at 4 mA cm^−2^ for CoMn-LDH, CoMn@CuZnS, and CoMn@CuZnFeS, respectively. The capacitance value dropped as the current density increased, and this decline in capacitance may be attributed to the equivalent acceleration limit of electron transport in the electrolyte ions D [49].

The CoMn-LDH sample exhibited excellent performance even at relatively high current densities. The encouraging electrochemical performance of CoMn-LDH may be attributed to the flex-type porous architecture, which enabled fast diffusion of electrolyte ions and a high surface area, which provided abundant reactive sites. Initially, when considering our first attempt to deposit CoMn@CuZnS and CoMn@CuZnFeS heterostructures, we were expecting improved electrochemical performance by the formation of CoMn@CuZnS and CoMn@CuZnFeS heterostructures, but both electrodes revealed a bit lower SC value than the pristine CoMn-LDH. This behavior could be correlated to the 3D-rod and granule formation, which resulted in a reduction in the total porosity and surface area compared to the CoMn-LDH structure. The reduced porosity decreased the rate of electrolyte ion transport and hindered the overall charge storage process. This can be overcome by the formation of more porous heterostructures with improved ion transport to achieve superior performance.

Compared to previous reports, the CoMn-LDH samples showed competitive three-electrode performance with C_A_ of 5.323 F cm^−2^, C of 279.49 mAh/g, and specific capacitance of Cs of 1829.42 F g^−1^ at 4 mA cm^−2^ (Table S3). In a previous work, CoMn-LDH was synthesized using a one-step electrodeposition process, yielding a Cs of 2465.2 F g^−1^ at 5 A g^−1^ [21]. Additionally, Jagadale et al. demonstrated electrodeposited growth of ultrathin nanoflakes of CoMn-LDH electrodes for supercapacitor applications, which showed a Cs of 875.3 F g^−1^ [24], whereas Chen et al. described the hydrothermal coating of CoMn-LDH nanowires and attained a Cs of 1192 F g^−1^ at 5 A g^−1^ [26]. Moreover, the hydrothermally deposited CoMn-LDH and CoMn-LDH/PPy were found to display lower capacitance at 2.5 A g^−1^ [51]. For CoMn-LDH grown in situ on flexible carbon fiber (CF) substrates, Zhao et al. obtained a Cs of 992 F g^−1^ at 4.2 A g^−1^ [52]. Meng et al. described that CoMnO nanowire arrays and NiMn-LDH exhibited C values of 95 and 90 mAh/g at 1 A g^−1^, respectively [53], and a Cs of 1369.75 F g^−1^ at 5 A g^−1^ was reported for CoMn-LDH nanoneedle arrays produced on NF substrates [45]. At 5 A g^−1^, the energy storage results of honeycomb-type NiCo-LDH revealed a Cs of 1175.1 F g^−1^ [54].

### 3.2. Fabrication and Testing of AC//CoMn-LDH HSC

**Figure 5:**
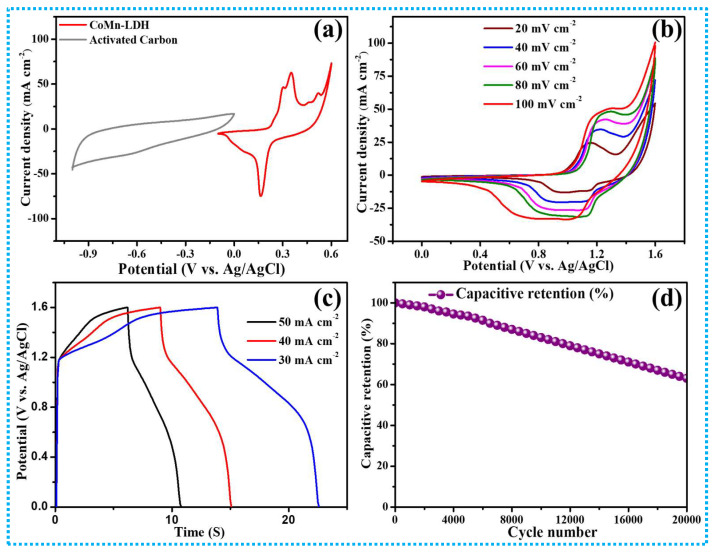
Electrochemical outcomes of AC//CoMn-LDH HSC device containing 4.0 M KOH electrolyte: (**a**) CV plots of distinct AC and CoMn-LDH at 5 mV s^−1^, (**b**) CV plots as a function of sweep rate, (**c**) GCD plots as a function of the current density, and (**d**) cycling stability of AC//CoMn-LDH HSC for 20,000 cycles.

The feasibility of the as-deposited CoMn-LDH electrodes for practical use was assessed by fabricating an HSC device. The HSC device was then tested by employing the anode (a high-power-density AC electrode) and cathode (a high-energy-density CoMn-LDH electrode) in 4 M KOH in a pouch cell configuration. In an HSC device, the operating voltage range should be greater than that of each electrode. Therefore, the potential range of the HSC was determined as the sum of the working voltages of the anode (0–1 V) and cathode (−0.1–0.6 V) at a sweep rate of 5 mV s^−1^ (Figure 5a). CV plots were obtained at operating voltages in the range of 1–1.6 V at 100 mV s^−1^, as displayed in Figure S2a, to determine the optimal potential. It was found that the CV plot at 1.6 V showed a higher area under the curve and had the correct potential window of the HSC device. The CV plots of the HSC device showed distinct redox peaks that signified promising rate capability and interfacial kinetics (Figure 5b). Similarly, the behavior of GCD plots was also assessed under different potential windows (Figure S2b). The GCD plots indicated a high-rate capacity and superior energy storage performance (Figure 5c). The calculated C_A_ values were found to be 781.25, 846.25, and 853.12 mF cm^−2^ for 50, 40, and 30 mA cm^−2^, respectively (Table 1). The C_A_ value of 853.12 mF cm^−2^ (C_s_ of 293.90 F g^−1^) attained for the AC//CoMn-LDH HSC device surpassed the capacity reported by HSC based on similar materials, namely CoMn-LDH//AC (23.5 F/g at 1 A/g) [24], Co-Mn LDH (65 F g^−1^ at 0.5 A g^−1^) [25], and CoMn-LDH/CFP (53.5 F g^−1^ at 0.5 A g^−1^) [55]. The developed HSC had a high PD of 250 W cm^−2^ at an ED of 0.347 Wh cm^−2^ and a noteworthy ED of 0.379 Wh cm^−2^ (130.62 Wh kg^−1^) at a PD of 150 W cm^−2^ (Table 1). The performance of this CoMn-LDH-based HSC approached or surpassed that of most previously reported HSCs, including CoMn-LDH (20.3 Wh kg^−1^ at 425 W kg^−1^) [25], CoMn-LDH//AC (362.3 W kg^−1^ at 34.5 Wh kg^−1^) [26], Co_0.2_Ni_0.8_LDH//AC (748.6 W kg^−1^ at 34.5 Wh kg^−1^) [56], NiCo LDH//AC (150 W kg^−1^ at 36.2 Wh kg^−1^) [57], CoFe LDH/LPG//AC (2800 W kg^−1^ at 25.7 Wh kg^−1^) [58], and NiCo-LDH@PANI@CC//AC (728 W kg^−1^ at 26.5 Wh kg^−1^) [59]. Capacitive retention of 63% was observed at 50 mA cm^−2^ for 20,000 GCD cycles (Figure 5d).

The cyclic stability results showed encouraging performance compared to previously reported devices, such as CoMn-LDH (83.7% retention over 3000 GCD cycles) [26], NiCo-LDH (70% retention over 10,000 GCD cycles) [10], Ni–Mn–S@NiCo_2_S_4_ (69.2% retention over 5000 GCD cycles) [13], CoMn-LDH/CFP (81% retention over 10,000 GCD cycles) [55], and Ni-Mn LDH/Co_3_O_4_ (70.9% retention over 5000 GCD cycles) [60]. Thus, the AC//CoMn-LDH HSC device results emphasize that CoMn-LDH is a high-performance material suitable for use in eco-friendly HSC devices. Table S4 compares the performance of different HSC devices based on LDH materials [21,25,44,52,55,60,61].

### 3.3. Electrocatalytic Performance of CoMn-LDH

**Figure 6:**
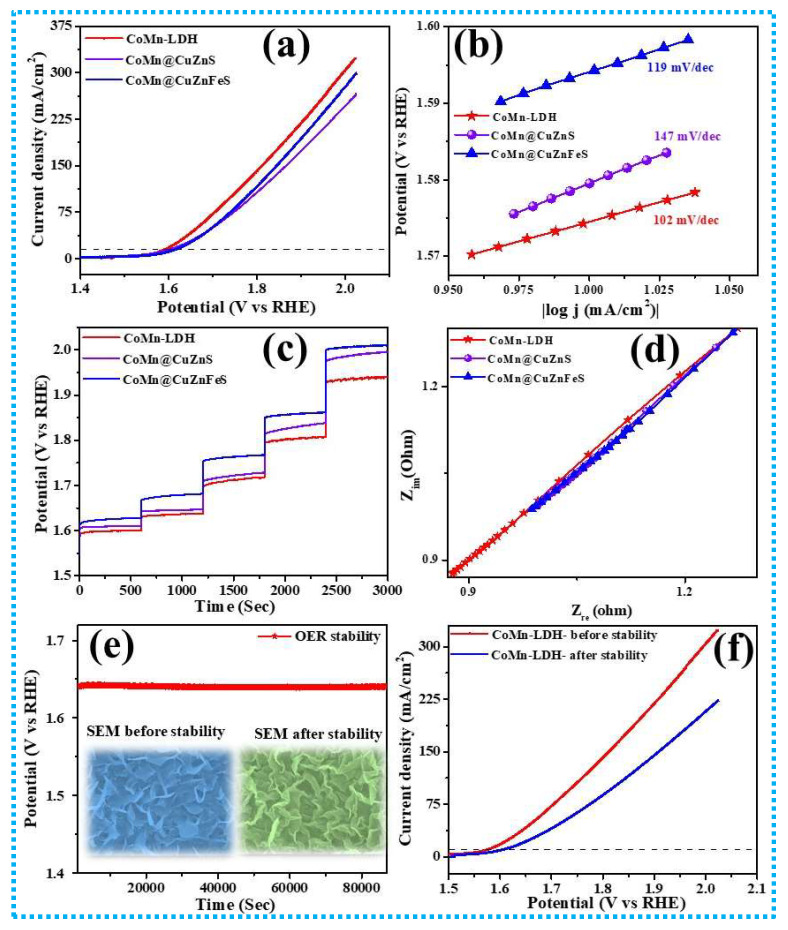
(**a**) OER LSV curves; (**b**) Tafel plots; (**c**) chronopotentiometric measurements of OER at different current densities; (**d**) Nyquist plots for CoMn-LDH, CoMn@CuZnS, and CoMn@CuZnFeS heterostructures in 1 M KOH solution; (**e**) chronopotentiometric stability study of CoMn-LDH (inset: corresponding SEM images before and after stability testing); and (**f**) LSV curves of CoMn-LDH before and after stability testing.

The catalytic process of all the electrodes was verified with a 1 M KOH electrolyte at 5 mV s^−1^ via a three-electrode setup. Figure 6a displays the polarization curve of all samples, providing further evidence that CoMn-LDH showed better OER performance. The CoMn-LDH electrode achieved a 370 mA cm^−2^ of current density (j) and required a low overpotential of 340 mV to attain j = 10 mA cm^−2^. The excellent OER activity of the CoMn-LH electrode can be credited to the ultralow thickness of the flex-type networks of nanosheets and the intrinsic layered structure. The heterostructures obtained comparatively low (j) of 299 and 267 mA cm^−2^ and request overpotentials of 350 and 366 mV, respectively. Interestingly, the nanogranular-type CoMn@CuZnFeS showed more encouraging catalytic activity than CoMn@CuZnS, which may be due to the introduction of Fe atoms [62]. However, overall, the inadequate performance of the heterostructured composites compared to the CoMn-LDH sample may be ascribed to the decreased porosity, which significantly reduced the active area, hindering the reaction kinetics and electron transport rate during HER activity. Generally, the CoMn-LDH-based OER catalysts showed moderately high potentials (η > 300 mV), indicating improved electrocatalytic OER activity. However, industrial applications demand achievement ≤ 100 mA cm^−2^ of a j at an overpotential of ≤350 mV, which remains challenging [2]. Notably, Table S5 reveals that CoMn-LDH showed comparable OER activity (340 mV) to achieve j = 10 mA cm^−2^ as previous reports for CoMn-LDH (300 mV) [63], Co_5_Mn-LDH/MWCNT (~300 mV) [64], Co_3_O_4_ nanomeshes (307 mV) [65], and CoMn-LDH/CNT (355 mV) [66].

To evaluate the OER kinetics, Tafel plots were obtained as follows [27]:

(10) ƞ=a+b logj  

where *a*, η, *b*, and *j* are the Tafel constant, overpotential, Tafel slope, and corresponding current density, respectively. Interestingly, CoMn-LDH realized a small Tafel slope (104 mV/dec) (Figure 6b) compared to CoMn@CuZnS (147 mV/dec) and CoMn@CuZnFeS (119 mV/dec) samples. A low Tafel slope demonstrates fast OER kinetics and a higher current density growth rate. Figure 6c reveals the chronopotentiometric graphs of all the electrodes at dissimilar j values. As displayed in Figure 6c, at each current step, the potential was approximately constant for 10 min, indicating the mechanical robustness and good mass transport of the synthesized electrodes. In addition, the electrode kinetics were studied using EIS measurements. The Nyquist plot showed a straight line, indicating a very low resistance associated with the electrodes (Figure 6d). CoMn-LDH exhibited the lowest R_ct_ (0.87 Ω), followed by CoMn@CuZnS (0.99 Ω) and CoMn@CuZnFeS (0.98 Ω), demonstrating rapid charge transfer kinetics. The EIS outcomes were consistent with the small Tafel slope and low overpotential obtained for CoMn-LDH and support its favorable electronic structure.

Long-term stability is vital for commercialization and must be evaluated to determine the superiority of an electrocatalyst for practical use. Figure 6e displays the results of chronopotentiometric measurements of the optimal CoMn-LDH electrodes at 20 mA cm^−2^ performed for 24 h. The potential remained unchanged for up to 3 h and then increased slightly. The cross-linked, flex-type networks of nanosheets contributed to the good stability, confirming that the created gas bubbles were released rapidly with no aggregation of bubbles on the anode’s surface. The polarization curve illustrated a reduction in the current density and an enhancement in the overpotential (370 mV) during continuous electrolysis for 24 h (Figure 6f). Furthermore, minor changes in the potential during the stability test and enhancement of the overpotential after the stability test can be credited to the shrinking and crumpled appearance of the morphology after long-term stability testing (Figure 6e inset). Long-term intercalation and deintercalation of the electrolyte ions are accountable for the formation of the shrinking and crumpled appearance of flex-type morphology after the stability test. It has been reported that sulfide-based heterostructures reveal strong chemisorption and effective catalytic conversion compared to the single-component materials [67].

**Figure 7:**
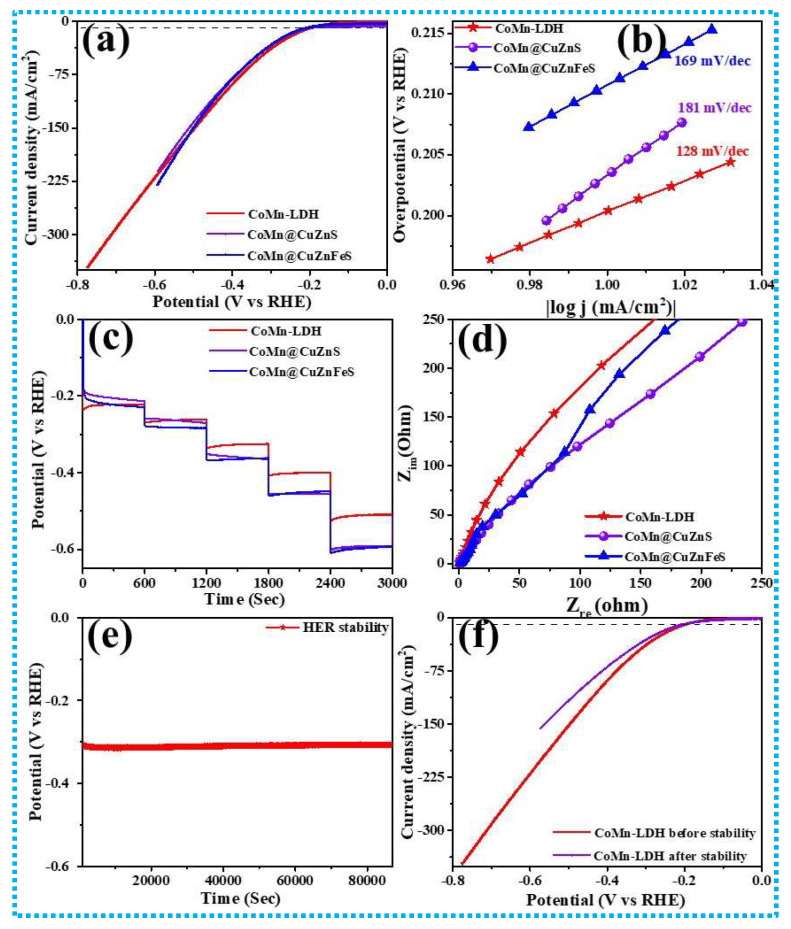
(**a**) HER LSV curves; (**b**) Tafel plots; (**c**) chronopotentiometric measurements of HER at different current densities; (**d**) Nyquist plots for CoMn-LDH, CoMn@CuZnS, and CoMn@CuZnFeS heterostructures in 1 M KOH solution; (**e**) chronopotentiometric stability study of CoMn-LDH; and (**f**) LSV curves of CoMn-LDH before and after stability testing.

Generally, HER catalytic activities are mainly determined by the intrinsic kinetics of nanomaterials. In addition to demonstrating encouraging OER performance, we tested HER activity of all the composite electrodes under similar measurement conditions. As expected, the polarization curve of CoMn-LDH displayed superior HER electrocatalytic activity (Figure 7a). The CoMn-LDH electrodes required a −199 mV overpotential to attain a j of 10 mA cm^−2^, which is significantly less than the CoMn@CuZnS (−215 mV) and CoMn@CuZnFeS (−222 mV). Furthermore, the Tafel slope was calculated using the Tafel equation [27] and was found to be 130, 196, and 160 mV/dec for CoMn-LDH, CoMn@CuZnS, and CoMn@CuZnFeS, respectively. The Tafel slope indicates that the Volmer–Heyrovsky step in HER on CoMn-LDH is the rate-determining step.

The electrocatalytic activity is governed by various parameters; however, the metal ion states and structural characteristics influence the electrocatalytic performance in the following manner. The electronic structure and redox characteristics of the metal ions in LDH materials and at the heterostructure interface significantly impact the electrocatalytic activity. In the CoMn-LDH material, multiple chemical states of Co (Co^3+^ and Co^2+^) and Mn (Mn^2+^, Mn^3+,^ and Mn^4+^) enable effective charge transportation during HER and OER activities. The synergetic influence of Co and Mn metal ions with different redox potentials can optimize the adsorption and desorption of reaction intermediates in HER and OER [68]. The vacancies and defects in the crystal structure form active sites with unique electronic properties. Moreover, LDH consists of metal hydroxide layers with interlayer anions and positive charges that balance the charges. These interlayers facilitate the diffusion of reactants and products within the LDH structure, and higher layer changes may increase the adsorption of electrolyte ions [68]. Figure 7b shows that a smaller Tafel slope demonstrates faster reaction kinetics and stronger catalytic activity. Figure 7c reveals the multistep chronopotentiometric curves for the electrodes at diverse current densities (−10 to −200 mA cm^−2^). All the catalysts displayed superior HER activity with the smallest potential response at each current step. Furthermore, the absence of noticeable variation in the HER potential over 600 s demonstrated good conductivity, robust mechanical properties, excellent stability, and typical mass transport behavior in the applied current density range. EIS measurements were performed to evaluate the interfacial characteristics and intrinsic charge transfer kinetics. Figure 7d shows Nyquist plots of the electrodes, revealing a much lower R_ct_ (0.84 Ω) among all other electrodes, suggesting a high charge transfer rate during the water dissociation process. Moreover, Table S6 compares the HER performance with previously reported studies.

The prolonged stability of HER activity is vital for the feasibility of CoMn-LDH in practical use. Therefore, chronopotentiometry measurements were taken at −20 mA cm^−2^ for 24 h, and the outcomes are shown in Figure 7e, which show outstanding durability of CoMn-LDH during the HER process, attributed to its unique structure. The minor change in the overpotential after continuous electrolysis for 24 h confirmed the excellent durability of the CoMn-LDH electrocatalyst in an alkaline solution. Furthermore, polarization plots were acquired before and after the stability tests and are presented in Figure 7f. The polarization graphs before and after the stability tests showed that the CoMn-LDH catalyst exhibited superior performance in an alkaline solution compared to CoMn@CuZnS and CoMn@CuZnFeS samples. As demonstrated in the Figure 6e inset, no significant changes occurred in the morphology after the extended stability test, indicating the robust durability of CoMn-LDH for catalytic applications.

## 4. Conclusions

We successfully fabricated CoMn-LDH, CoMn@CuZnS, and CoMn@CuZnFeS heterostructures as a bifunctional electrode for energy storage and an electrocatalyst for water splitting. CoMn-LDH demonstrated outstanding energy storage capacity (C_A_ of 5.323 F cm^−2^), excellent rate capability, and high diffusion coefficients, with dominant battery-type behavior. The remarkable electrochemical performance of CoMn-LDH can be ascribed to its architecture of self-assembled, cross-linked, flexible nanosheets, which offers abundant active sites and high contact areas with electrolytes, enabling efficient intercalation and deintercalation of electrolyte ions. On the other hand, CoMn@CuZnS and CoMn@CuZnFeS heterostructures revealed poor areal and specific capacity of 3.047 F cm^−2^ (150.17 mAh/g) and 2.210 F cm^−2^ (105.22 mAh/g), respectively. The heterostructure’s low performance can be credited to the reduced porosity and surface area, which effectively blocked the electrolyte ion’s transportation path and hindered electrochemical performance. Moreover, the as-fabricated AC//CoMn-LDH HSC device delivered high energy storage capacity with better durability (63% capacity perseverance) over 20,000 cycles. Further, CoMn-LDH electrodes demonstrated better OER and HER performance with small Tafel slopes of 102 and 128 mV/dec compared to heterostructures in an alkaline electrolyte. The CoMn@CuZnS and CoMn@CuZnFeS electrodes depicted even larger Tafel slopes of 147 and 119 mV/dec and 181 and 169 mV/dec for OER and HER processes, respectively. Thus, our findings open new ideas to endow other transitional metal materials for a variety of bifunctional high-tech applications, including efficient energy conversion and storage devices.

## Figures and Tables

**Table 1:** The supercapacitive parameters of the AC//CoMn-LDH HSC device.

Sr. No.	Current Density (mA cm^−2^)	Areal Capacitance (C_A_) mFcm^−2^	Specific Capacitance (C_S_) F g^−1^	Specific Capacity mAh/g	Energy Density Wh cm^−2^	Power Density W cm^−2^
1	30	853.12	293.90	130.62	0.347	250
2	40	846.25	291.53	129.57	0.376	200
3	50	781.25	269.14	119.62	0.379	150

## Data Availability

The original contributions presented in the study are included in the article; further inquiries can be directed to the corresponding authors.

## Supplementary Materials

The following supporting information can be downloaded at https://www.mdpi.com/article/10.3390/ma18030604/s1, Material characterization; electrochemical evaluation; fabrication of hybrid supercapacitor device; Table S1: The surface area, average pore volume, and pore size acquired from BET analysis of CoMn-LDH, CoMn@CuZnS, and CoMn@CuZnFeS heterostructures; Figure S1: (a–c) EDS spectrographs and charge storage contribution; (d) potential window variation of CoMn-LDH electrodes; and (e–g) charge contribution for CoMn-LDH, CoMn@CuZnS, and CoMn@CuZnFeS heterostructures; Table S2: The different electrochemical parameters for CoMn-LDH, CoMn@CuZnS, and CoMn@CuZnFeS heterostructured electrodes; Table S3: Comparison of CoMn-LDH electrode’s electrochemical performance with recently reported materials; Figure S2: (a) CV plots at different potential windows at 100 mV s^−1^ and (b) GCD and CV plots at different potential windows at 100 mV s^−1^ of the AC//CoMn-LDH HSC device; Table S4: Supercapacitive performance comparison of different HSCs based on LDH materials; Table S5: OER performance comparison with previously reported materials; Table S6: HER performance comparison with previously reported materials. References [69,70,71,72,73,74,75,76,77,78] are cited in the supplementary materials.
