# Supplementary material for: Hierarchical CoMn-LDH and Heterostructured Composites for Advanced Supercapacitors and Electrocatalysis Applications
Source: Materials (Basel). 2025 Jan 28;18(3):604. doi: 10.3390/ma18030604 (PMC11818902; doi:10.3390/ma18030604)
Supplement: Supplementary file 1 [file materials-18-00604-s001.zip › materials-3275518-supplementary.pdf]

Supplementary Material

Hierarchical CoMn-LDH and Heterostructured Composites for Advanced Supercapacitors and Electrocatalysis Applications

Ganesh T. Chavan [1], Deepak P. Dubal [2], Pritam J. Morankar [3], Chan-Wook Jeon [3], Jinsung An [1] and Ki-Han Song [4,*]

[1] Department of Civil & Environmental Engineering, Hanyang University ERICA, Ansan 15588, Republic of Korea; gtchavan1992@gmail.com (G.T.C.); jsan86@hanyang.ac.kr (J.A.)
[2] Centre for Materials Science, School of Chemistry and Physics, Queensland University of Technology (QUT), 2 George Street, Brisben, QLD 4000, Australia
[3] School of Chemical Engineering, Yeungnam University, 280 Daehak-ro, Gyeongsan 38541, Republic of Korea
[4] Department of Civil Engineering, Seoul National University of Science and Technology, Seoul 01811, Republic of Korea
* Correspondence: kihansong@seoultech.ac.kr

1. Materials characterizations

1.1. Physical characterizations

The crystal structure of all the samples was determined by X-ray diffraction (X'pert Pro, Panalytical) with Cu Kα radiation at λ = 1.5406 Å. X-ray photoelectron spectroscopy (XPS, K-alfa, Thermo Scientific) was used to determine the chemical states. Field-emission scanning electron microscopy (FESEM; Hitachi S-4800, Hitachi, Japan) with elemental dispersive spectroscopy (EDS) was employed to identify the surface morphologies of the materials. High-resolution transmission electron microscopy (Tecnai G2 F20 S-TWIN) was used to examine the nanostructures of the materials.

1.2. Electrochemical evaluation

Electrochemical supercapacitive experiments were performed in a 4 M KOH electrolyte using a battery cycler (Biologic Instrument-WBCS3000) with a three-electrode configuration. Electrocatalytic measurements were performed using a 1 M KOH electrolyte in the same configuration. The specific capacity (C), capacitance (C_s), ED, and PD were determined using standard equations [1-5]:

$$C_A\ (F\ cm^{-2}) = \frac{I_d \times T_d}{A \times dV} \quad \text{..... (1)}$$

$$C\ (mAh/g) = \frac{I \times T_d}{3.6 \times m} \quad \text{..... (2)}$$

$$C_s\ (F\ g^{-1}) = \frac{I_d \times T_d}{m \times dV} \quad \text{..... (3)}$$

$$ED\ (Wh\ kg^{-1}) = \frac{C_s \times (dV^2)}{7.2} \quad \text{..... (4)}$$

$$PD\ (W\ kg^{-1}) = \frac{Ed\ x\ 3600}{T_d} \quad \text{..... (5)}$$

where C, C_A, and C_S are the specific capacity, areal capacitance, and specific capacitances, I_d is the discharge current, T_d is the discharge time, A is the active area (1 cm^2), dV is the potential window, Ed is the energy density, and m is the active mass.

1.3. Fabrication of AC//CoMn-LDH hydride supercapacitor device (HSC)

The AC//CoMn-LDH asymmetric hydride supercapacitor cell (HSC) was fabricated as follows:

The activated carbon electrode (AC) anode was obtained by combining 10 wt% acetylene black, 80 wt% AC, and 10 wt% polyvinylidene fluoride in a homogeneous slurry. Then, this slurry was deposited on the NF substrates and dehydrated at 80 °C.

The as-deposited CoMn-LDH electrode served as the cathode, while the activated carbon (AC) electrode acted as the anode. To achieve charge balance between the CoMn-LDH (q^+) and AC (q^-) electrodes for HSC device fabrication, the mass-loading ratio was estimated as follows [1]:

$$\frac{M_+}{M_-} = \frac{C_- \times \Delta V_-}{C_+ \times \Delta V_+} \quad \text{..... (5)}$$

where M_- and M_+ are the masses of the anode and the cathode, respectively, C_- and C_+ are the specific capacitances of the anode and cathode, respectively, and V is the voltage range.

Table S1. The surface area, average pore volume, and pore size acquired from BET analysis of CoMn-LDH, CoMn@CuZnS, and CoMn@CuZnFeS heterostructures.

Electrocatalyst	Surface area (m^2/g)		Pore size (nm)	Average pore volume (cm^3/g)
	Single point surface area at P/P^0= 0.269868445	BET surface area		
CoMn-LDH	0.9889	16.11	12.34	0.04972
CoMn@CuZnS	0.9900	8.82	16.81	0.02082
CoMn@CuZnFeS	0.9904	12.62	18.52	0.05844

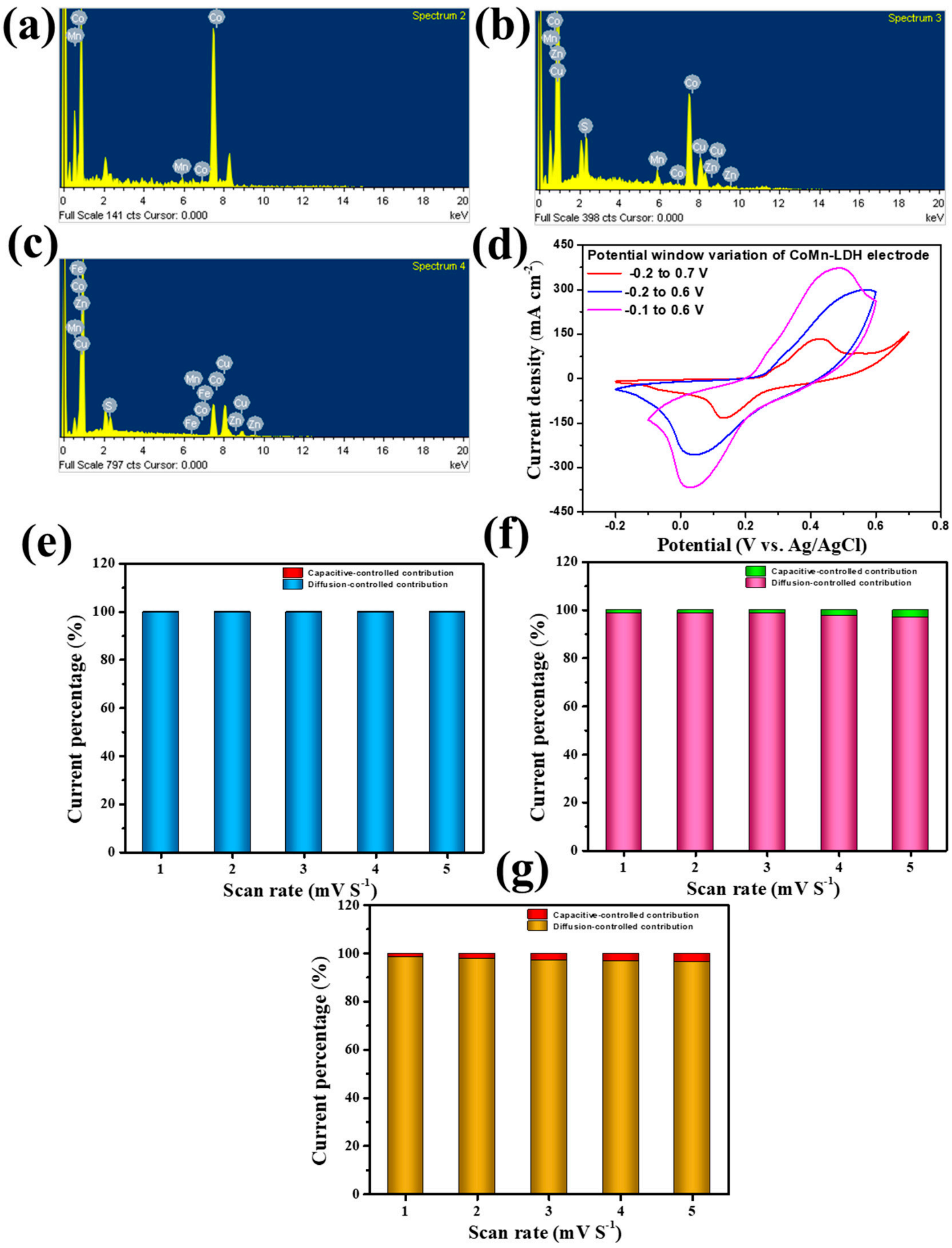

Figure S1. (a-c) The EDS spectrographs and charge storage contribution, (d) potential window variation of CoMn-LDH electrodes, and (e-g) charge contribution for CoMn-LDH, CoMn@CuZnS, and CoMn@CuZnFeS heterostructures.

Table S2. The different electrochemical parameters for CoMn-LDH, CoMn@CuZnS, and CoMn@CuZnFeS heterostructured electrodes.

Sr. No.	Sample code	Current density (mA cm^{-2})	Active mass (mg)	Discharge time (sec)	Areal capacitance (F cm^{-2})	Specific capacitance (F g^{-1})
1	CoMn-LDH	4	2.99	732	5.323	1829.42
2	CoMn@CuZnS	4	3.10	419	3.047	983
3	CoMn@CuZnFeS	4	3.21	304	2.210	688.75

Table S3. Comparison of CoMn-LDH electrode's electrochemical performance with recently reported materials.

Material	Synthesis method	Electrolyte	Specific Capacitance (F g^{-1})	Ref.
Co-Mn LDH	Electrodeposition	2 M KOH	2673.6	6
Co-Mn LDH	Solvothermal and Hydrothermal	1 M KOH	318	7
Mn-Co-Fe	electrodeposition	6 M KOH	1200	8
Co-Mn LDH	In-situ growth	PVA/LiOH	1079	9
CoMn LDH/CFP	Electrodeposition	1 M KOH	980	10
Ni-Mn LDH/Co_3O_4	Hydrothermal	1 M KOH	1327	11
Co_3O_4@glucose	Hydrothermal	6 M KOH	1644	12
$Ni_{0.6-x}Mo_{0.4-x}Ir_x$-oxide	Chemical	6 M KOH	108 mF cm^{-2}	13
Ru/RuO_2	Hydrothermal	PAM-KOH gel	1297 mF cm^{-2}	14
MnO_2	Electrodeposition	1.0 M Na_2SO_4	469	15
NiO	Hydrothermal	1 M KOH	132	16
IrO_2	Electrospinning	1 M Na_2SO_4	705	17
**CoMn-LDH**	**Hydrothermal**	**2 M KOH**	**1829.42**	**Present work**

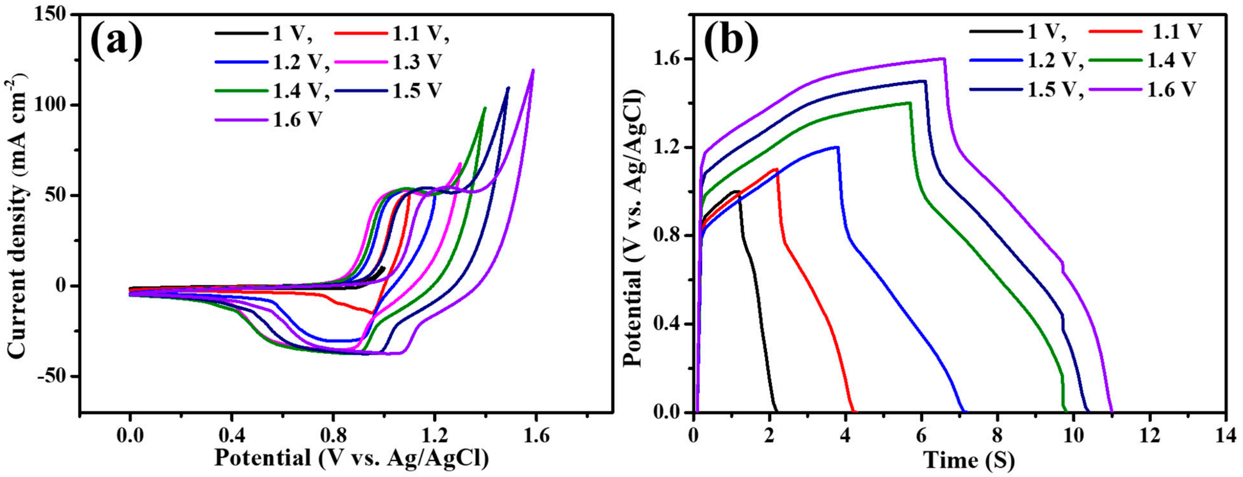

Figure S2. (a) The CV plots at different potential windows at 100 mV s⁻¹ and (b) GCD plots at CV plots at different potential windows at 100 mV s⁻¹ of the AC//CoMn-LDH HSC device.

Table S4. The supercapacitive performance comparison of different HSCs based on LDH materials.

Material	Synthesis method	Morphology	Electrolyte	Specific Capacitance ($F\ g^{-1}$)	Number of cycles	Capacity retention (%)	Energy density ($Wh\ kg^{-1}$)	Power Density ($W\ kg^{-1}$)	Ref.
Co-Mn LDH	Electrodeposition	Thin layer structure	2 M KOH	274.26	5,000	89.2	97.5	8000.0	6
Co-Mn LDH	Solvothermal and Hydrothermal	Flakes	1 M KOH	0.65	10,000	99.7	20.3	435	7
Mn-Co-Fe	electrodeposition	Nano-platelets	6 M KOH	36.5	4,000	96	11.4	1125	8
Co-Mn LDH	In-situ growth	Nano sheet	PVA/LiOH	-	6,000	88.3	126.1	65.6	9
CoMn LDH/CFP	Electrodeposition	Nano sheet	1 M KOH	53.5	10,000	-	19.1	400	10
Ni-Mn LDH/Co_3O_4	Hydrothermal	Nano needle	1 M KOH	79.7	5,000	70.9	35.87	449.99	11
Co_3O_4@glucose	Hydrothermal	Nanowires	6 M KOH	108.4	2000	89.1	38.4	800	12
**CoMn-LDH**	**Hydrothermal**	**Cross-linked flex**	**2 M KOH**	**293.90**	**20,000**	**63**	**0.379 $Wh\ cm^{-2}$**	**250 $W\ cm^{-2}$**	**Present work**

Table S5. The OER performance comparison with previously reported materials.

Material	Synthesis method	Morphology	Electrolyte	OER overpotential (mV)	OER Tafel (mV dec^{-1})	Current density (mA cm^{-2})	Ref
CoMn-LDH	Hydothermal	Pine-needle-shaped nanowires	1 M KOH	300	126	30	18
Co_5Mn-LDH/ MWCNT	Chemical	Sheetlike	1 M KOH	300	73.6	10	19
Co_3O_4	Chemical	Flower-like	1 M KOH	307	76	10	20
CoMn-LDH/CNT	Co-precipitation	Nanoplates	0.1 M KOH	355	45	10	21
Co_8Mn_2-LDH	Hydrothermal	Nanosheets	1 M KOH	307	86	10	22
CoMo-LDH	Co-precipitation	Ultrathin nanosheet	1 M KOH	300	56	10	23
**CoMn-LDH**	**Hydrothermal**	**Cross-linked flex**	**1 M KOH**	**340**	**104**	**10**	**Current work**

Table S6. The HER performance comparison with previously reported materials.

Material	Synthesis method	Morphology	Electrolyte	HER overpotential (mV)	HER Tafel (mV dec^{-1})	Current density (mA cm^{-2})	Ref
CoMn LDH@Cu(OH)$_2$/CF	Hydrothermal	Nanorods	1 M KOH	155	146.7	10	24
CoMn-LDH	Unipolar pulse electrodeposition	Nanosheet arrays	1 M KOH	230	219	10	25
NiFe-LDH	Polyol-assisted reduction process	Nanosheets	1 M KOH	245	194.2	10	26
CoMoV	Hydrothermal	Nanosheets	1 M KOH	150	-	10	27
Mn–O/Co–P/CC	facile grown	Nanohybrid nanosheet	1 M KOH	150	78	10	28
NiFe-LDH	Electrodeposition	Nanosheets	1 M KOH	272	101	10	29
**CoMn-LDH**	**Hydrothermal**	**Cross-linked flex**	**1 M KOH**	**199**	**130**	**10**	**Current work**

References

[1] Chavan, G.T.; Amate, R.U.; Lee, H.; Syed, A.; Bahkali, A.H.; Elgorban, A.M.; Jeon, C.W.; Rational design of 3D hollow cube architecture for next-generation efficient aqueous asymmetric supercapacitors, *J. Energy Storage*, **2023**, *61*, 106757. https://doi.org/10.1016/j.est.2023.106757.

[2] Ahmed, N.; Ali, B.A.; Allam, N.K.; Optimized electrosynthesis approach of Manganese-Nickel- Cobalt chalcogenide nanosheet arrays as binder-free battery materials for asymmetric electrochemical supercapacitors, *Electrochim. Acta*. **2021**, 396, 139191. https://doi.org/10.1016/j.electacta.2021.139191.

[3] Raju, T.D.; Gopalakrishnan, A.; Badhulika, S.; Facile synthesis of 3D/2D Cu2Se cauliflower/CuS nanosheets composite as a binder-free electrode for high-performance asymmetric solid-state supercapacitors, *J. Alloys Compd*. **2020**, *845*, 156241. https://doi.org/10.1016/j.jallcom.2020.156241

[4] Conway, B.; Electrochemical Supercapacitors Scientific Fundamentals and Technological Applications, Kluwer acadamic/ plenum publisher, 1999.

[5] Khaladkar, S.R.; Maurya, O.; Gund, G.; Sinha, B.; Dubal, D.; Deshmukh, R.R.; Kalekar, A.; *J. Energy Chem*. **2023**, *87*, 304-313. https://doi.org/10.1016/j.jechem.2023.08.048

[6] Emin, A.; Song, X.; Du, Y.; Chen, Y.; Yang, M.; Zou, S.; Fu, Y.; Li, J.; Li, Y.; He, D.; One-step electrodeposited Co and Mn layered double hydroxides on Ni foam for high-performance aqueous asymmetric supercapacitors, *J. Energy Storage*, **2022**, *50*, 104667. https://doi.org/10.1016/j.est.2022.104667.

[7] Ochai-Ejeh, F.O.; Madito, M.J.; Momodu, D.Y.; Khaleed, A.A.; Olaniyan, O.; Manyala, N.; High performance hybrid supercapacitor device based on cobalt manganese layered double hydroxide and activated carbon derived from cork (Quercus Suber), *Electrochim. Acta*. **2017**, *252*, 41–54, https://doi.org/10.1016/j.electacta.2017.08.163.

[8] Elkholy, A.E.; El-Taib Heakal, F.; Allam, N.K.; A facile electrosynthesis approach of amorphous Mn-Co-Fe ternary hydroxides as binder-free active electrode materials for high-performance supercapacitors, *Electrochim. Acta*. **2019**, *296*, 59–68, https://doi.org/10.1016/j.electacta.2018.11.038.

[9] Zhao, J.; Chen, J.; Xu, S.; Shao, M.; Yan, D.; Wei, M.; Evans, D.G.; Duan, X.; CoMn-layered double hydroxide nanowalls supported on carbon fibers for high-performance flexible energy storage devices, *J. Mater. Chem. A*. **2023**, *1*, 8836–8843. https://doi.org/10.1039/c3ta11452j.

[10] Zhao, C.; Tian, S.; Nie, P.; Deng, T.; Ren, F.; Chang, L.; Electrodeposited binder-free CoMn LDH/CFP electrode with high electrochemical performance for asymmetric supercapacitor, *Ionics*, **2020**, *26*, 1389–1396. https://doi.org/10.1007/s11581-019-03290-0.

[11] Ouyang, L.; Hsiao, C.H.; Chen, Y.C.; Lee, C.Y.; Tai, N.H.; Fabrication of Ni-Mn LDH/Co_3O_4 on carbon paper for the application in supercapacitors, *Surf. Interfaces*, **2022**, *28*, 101574. https://doi.org/10.1016/j.surfin.2021.101574.

[12] Quan, W.; Xu, Y.; Wang, Y.; Meng, S.; Jiang, D.; Chen, M.; Hierarchically structured Co_3O_4@glucose-modified LDH architectures for high-performance supercapacitors, *Appl. Surf. Sci*. **2019**, *488*, 639–647. https://doi.org/10.1016/j.apsusc.2019.05.301.

[13] Vidales, A.G,; Kim, J.; Omanovic, S.; $Ni_{0.6-x}Mo_{0.4-x}Ir_x$-oxide as an electrode material for supercapacitors: investigation of the influence of iridium content on the charge storage/delivery, *J. Solid State Electrochem.* **2019**, *23*, 2129-2139, https://doi.org/10.1007/s10008-019-04311-8.

[14] Kumar, M.; Kar, K.K.; Paik, P.; Supercapacitor electrodes based on Ru/RuO_2 decorated on N,S-doped few-layer graphene, *Chem. Eng. J.* **2024**, 499, 156414, https://doi.org/10.1016/j.cej.2024.156414

[15] Ming, Z.; Yan, C.; Dingyu, Y.; Jitao, L.; High performance MnO_2 supercapacitor material prepared by modified electrodeposition method with different electrodeposition voltages, J. Energy Storage, **2020**, *29*, 101363, https://doi.org/10.1016/j.est.2020.101363.

[16] Dhas, S.D.; Maldar, P.S.; Patil, M.D.; Nagare, A.B.; Waikar, M.R.; Sonkawade, R.G.; Moholkar, A.V.; Synthesis of NiO nanoparticles for supercapacitor application as an efficient electrode material, Vacuum, **2020**, *181*, 109646, https://doi.org/10.1016/j.vacuum.2020.109646.

[17] Beknalkar, S.A.; Teli, A.M.; Harale, N.S.; Patil, D.S.; Pawar, S.A.; Shin, J.C.; Patil, P.S.; Fabrication of high energy density supercapacitor device based on hollow iridium oxide nanofibers by single nozzle electrospinning, *Appl. Surf. Sci*. **2021**, *546*, 149102, https://doi.org/10.1016/j.apsusc.2021.149102.

[18] Li, X.; Patil, K.; Agarwal, A.; Babar, P.; Jang, J.S.; Chen, X.; Yoo, Y.T.; Kim, J.H.; $Ni(OH)_2$Coated CoMn-layered double hydroxide nanowires as efficient water oxidation electrocatalysts, *New J. Chem*. **2022**, *46*, 2044–2052. https://doi.org/10.1039/d1nj04792b.

[19] Jia, G.; Hu, Y.; Qian, Q.; Yao, Y.; Zhang, S.; Li, Z.; Zou, Z.; Formation of Hierarchical Structure Composed of (Co/Ni)Mn-LDH Nanosheets on MWCNT Backbones for Efficient Electrocatalytic Water Oxidation, *ACS Appl. Mater. Interfaces*. **2016**, *8*, 14527–14534. https://doi.org/10.1021/acsami.6b02733.
[20] Li, Y.; Li, F.M.; Meng, X.Y.; Li, S.N.; Zeng, J.H.; Chen, Y.; Ultrathin Co_3O_4 Nanomeshes for the Oxygen Evolution Reaction, *ACS Catal*. **2018**, *8*, 1913–1920, https://doi.org/10.1021/acscatal.7b03949.
[21] Liu, Z.; Yu, C.; Han, X.; Yang, J.; Zhao, C.; Huang, H.; Qiu, J.; CoMn Layered Double Hydroxides/Carbon Nanotubes Architectures as High-Performance Electrocatalysts for the Oxygen Evolution Reaction, *ChemElectroChem*. 2016, 3, 906–912. https://doi.org/10.1002/celc.201600116.
[22] Pan, S.; Li, B.; Yu, J.; Zhao, L.; Zhang, Y.; Composition controllable fabrication of ultrathin 2D CoMn layered double hydroxides for highly efficient electrocatalytic oxygen evolution, *Appl. Surf. Sci*. **2021**, *539*, 148305, https://doi.org/10.1016/j.apsusc.2020.148305.
[23] Bao, J.; Wang, Z.; Xie, J.; Xu, L.; Lei, F.; Guan, M.; Hunag, Y.; Zhao, Y.; Xia, J.; Li, H.; The CoMo-LDH ultrathin nanosheet as a highly active and bifunctional electrocatalyst for overall water splitting, *Inorg. Chem. Front.*, **2018**, 5, 2964-2970, https://doi.org/10.1039/C8QI00867A.
[24] Wang, J.; Yu, H.; Wang, X.; Chen, C.; Li, S.; Cai, N.; Chen, W.; Xue, Y.; Li, H.; Yu, F.; Tri-metal-based hollow nanorods-on-microrod arrays as efficient water splitting electrocatalysts, *J. Indust. Eng. Chem*. **2022**, *105*, 427-434, https://doi.org/10.1016/j.jiec.2021.10.007.
[25] Sirisomboonchai, S.; Kitiphatpiboon, N.; Chen, M.; Li, S.; Li, X.; Kongparakul, S.; Samart, C.; Zhang, L.; Abudula, A.; Guan, G.; Multi-Hierarchical Porous Mn-Doped CoP Catalyst on Nickel Phosphide Foam for Hydrogen Evolution Reaction, *ACS Appl. Energy Mater*. **2022**, *5*, 149–158, https://doi.org/10.1021/acsaem.1c02650.
[26] Zhou, Y.; Wang, Z.; Pan, Z.; Liu, L.; Xi, J.; Luo, X.; Shen, Y.; Exceptional Performance of Hierarchical Ni–Fe (hydr)oxide@NiCu Electrocatalysts for Water Splitting, *Adv. Mat*. **2019**, *31*, 1806769, https://doi.org/10.1002/adma.201806769.
[27] Bao, J.; Wang, Z.; Xie, J.; Xu, L.; Lei, F.; Guan, M.; Zhao, Y.; Huang, Y.; Li, H.; A ternary cobalt–molybdenum–vanadium layered double hydroxide nanosheet array as an efficient bifunctional electrocatalyst for overall water splitting, *Chem. Commun.*, **2019**, *55*, 3521-3524, https://doi.org/10.1039/C9CC00269C.
[28] Zhou, D.; Wang, Z.; Long, X.; An, Y.; Lin, H.; Xing, Z.; Ma, M.; Yang, S.; One-pot synthesis of manganese oxides and cobalt phosphides nanohybrids with abundant heterointerfaces in an amorphous matrix for efficient hydrogen evolution in alkaline solution, *J. Mater. Chem. A*, **2019**, *7*, 22530-22538, https://doi.org/10.1039/C9TA07601H.
[29] Zhai, P.; Xia, M.; Wu, Y.; Zhang, G.; Gao, J.; Zhnag, B.; Cao, S.; Zhang, Y.; Li, Z.; Fan, Z.; Wang, C.; Zhang, X.; Miller, J.T.; Sun, L.; Hou, J.; Engineering single-atomic ruthenium catalytic sites on defective nickel-iron layered double hydroxide for overall water splitting, *Nat. Commun.* **2021,** *12*, 4587, https://doi.org/10.1038/s41467-021-24828-9.
